# Case report: Can cochlear implant stimulation lead to improved balance even after vestibular neurectomy?

**DOI:** 10.3389/fneur.2023.1248715

**Published:** 2023-08-25

**Authors:** Morgana Sluydts, Chloë De Laet, Liesbeth De Coninck, Catherine Blaivie, Joost J. S. van Dinther, Erwin Offeciers, Floris L. Wuyts, Andrzej Zarowski

**Affiliations:** ^1^Ear-Nose-Throat Department, European Institute for Otorhinolaryngology, Head and Neck Surgery, Antwerp, Belgium; ^2^Lab for Equilibrium Investigations and Aerospace, Faculty of Sciences, University of Antwerp, Antwerp, Belgium

**Keywords:** vestibular co-stimulation, cochlear implant, bilateral vestibulopathy, vestibular neurectomy, tinnitus suppression

## Abstract

**Introduction:**

In a previous manuscript from our research group, the concept of vestibular co-stimulation was investigated in adult subjects who received a cochlear implant (CI). Despite what literature reports state, no signs of vestibular co-stimulation could be observed.

**Results:**

In this case report, it was described how a woman, who previously underwent a neurectomy of the left vestibular nerve and suffers from bilateral vestibulopathy (BVP), reported improved balance whenever her CI on the left was stimulating. Unexpectedly, the sway analyses during posturography indeed showed a clinically relevant improvement when the CI was activated.

**Discussion:**

Vestibular co-stimulation as a side effect of CI stimulation could not be the explanation in this case due to the ipsilateral vestibular neurectomy. It is more likely that the results can be attributed to the electrically restored auditory input, which serves as an external reference for maintaining balance and spatial orientation. In addition, this patient experienced disturbing tinnitus whenever her CI was deactivated. It is thus plausible that the tinnitus increased her cognitive load, which was already increased because of the BVP, leading to an increased imbalance in the absence of CI stimulation.

## 1. Introduction

Vestibular co-stimulation is a phenomenon that occurs when currents delivered by a cochlear implant (CI) spread from the cochlea toward the surrounding vestibular neural structures and tissues. Multiple reports of vestibular co-stimulation have been made throughout the years. In 1978, Black et al. (1) reported that the vestibulospinal control in patients fitted with a CI was disturbed during electrical CI stimulation. Eisenberg et al. (2) investigated the possible detrimental effect of a single-electrode CI on the vestibular system. However, they concluded that the postural stability improved. Later, Bance et al. (3) observed an electrically evoked nystagmus through electrical auditory nerve stimulation delivered by a multichannel CI. In several more recent studies, the effect of the CI on different vestibular reflexes was observed, e.g., increased gains of the video Head Impulse Test (vHIT) (4), improved postural stability and gait (5–8), improved perception of verticality, and electrically evocable cervical and ocular vestibular-evoked myogenic potentials (9, 10). In these studies, it was often hypothesized that the findings resulted from vestibular co-stimulation.

An alternative approach for explaining the observed postural improvements suggests that the restored auditory cues are the underlying mechanism. Zhong and Yost (11) hypothesized that auditory cues act as an external reference for maintaining balance. For patients who cannot (fully) perceive environmental sounds due to hearing loss, it can thus be hypothesized that they have more difficulty in maintaining balance, regardless of the status of the peripheral vestibular function. This hypothesis was supported by the study of Stevens et al. (12) and Shayman et al. (13) who reported improved (decreased) postural sway and gait (respectively) when the auditory input was provided in different normal hearing patients with or without vestibular loss (12) and patients with or without CI or hearing aids (13).

In a previous manuscript from our research group, the concept of vestibular co-stimulation was investigated in four adult subjects who received a CI (14). However, no signs of vestibular co-stimulation could be observed. It was therefore concluded that vestibular co-stimulation, as a form of far-field electrical stimulation, is unlikely to functionally improve balance. After the completion of this pilot study, one additional patient explicitly reported that her balance improves when she activates her CI. This subject, however, previously underwent a vestibular neurectomy (elsewhere), which means that accidental co-stimulation of the vestibular nerve was impossible. In order to further understand how postural balance can improve in a CI patient after vestibular neurectomy, this patient was invited to evaluate the effect of CI stimulation on static and dynamic posture.

## 2. Case description

### 2.1. Case history

In 2002, a female patient (61 years) presented with vertigo attacks and was diagnosed with Meniere's disease (MD) (Table 1). In 2009, a neurectomy of the left vestibular nerve was performed elsewhere. This procedure eliminated severe vertigo attacks but induced chronic imbalance in combination with left-sided hearing loss and ipsilateral pulsatile tinnitus.

**Table 1:** Patient demographics.

Age at implantation	59 years
Age at time of the study	61 years
Diagnosis	Right: Uncompensated acute unilateral vestibulopathy (probably vestibular neuritis) Left: Definite unilateral Meniere's disease (vestibular neurectomy in 2009 elsewhere)
PTA (dB HL) (average 500, 1,000, 2,000, 4,000 Hz)	Right: 8 dB HL Left: 96 dB HL (dd. 2018) (unaided)
Implant side	Left
Implant type	FlexSoft28 (Med-El, Innsbruck, Austria)
Contralateral ear	Normal hearing

CI, cochlear implant; PTA, pure tone audiometry; dB HL, decibel hearing level.

In 2013, this patient consulted one of the vestibular physicians at the European Institute for Otorhinolaryngology (Antwerp, Belgium).

She presented with chronic symptoms of imbalance, oscillopsia, lightheadedness, visually induced dizziness, fatigue, left-sided pulsatile tinnitus, aural fulness, and hearing loss. Walking in darkness or on uneven surfaces increased her imbalance, which is typically seen in patients with bilateral vestibulopathy (BVP) (15). The contralaterally reduced vestibular functions were probably caused by vestibular neuritis, a diagnosis which was based on magnetic resonance imaging (MRI, in 2017) that showed decreased signal intensity and decreased fluid in the superior semicircular canal on the right, which could be a possible sequela of labyrinthitis. The objective test results of the vHIT (overt and covert saccades and gains below 0.60 for all canals), caloric test (total caloric sum = 0°/s), and rotational test (gain = 0%), which were obtained in 2016 (so prior to the respective MRI) clearly showed that the contralateral (right) function was also impaired (for methodological test details see Section 2.2). However, as hearing on the right did not deteriorate at any point in time, it was hypothesized that a previous right-sided episode of vestibular neuritis (instead of complete labyrinthitis) was more likely.

Later, in 2017, the patient started having difficulty with concentration, short-term memory, and spatial orientation. No abnormalities were found during the neurological assessment. The vestibular examination confirmed BVP (15) with a first-degree spontaneous nystagmus to the right.

The vestibular neurectomy successfully ceased the vertigo attacks, but aural fullness and tinnitus remained as the cochlear nerve was still intact. Unfortunately, no pre- and post-operative vestibular function tests were available as the neurectomy was performed elsewhere.

In 2016, intratympanic injections (dexamethasone) were performed in an attempt to stop or reduce tinnitus, unfortunately without effect. Because the patient continued to experience debilitating tinnitus, cochlear implantation was finally performed on the left side in 2018, with tinnitus suppression as the main goal. Prior to the implantation, round window stimulation under local anesthesia verified that this was possible in this patient (16, 17). The activation of the CI successfully suppressed the tinnitus, but it returned upon device deactivation.

Later, in 2022, during an annual fitting, the patient reported that her balance markedly improved whenever the CI was activated. No spontaneous nystagmus was observed with or without CI stimulation after the implantation. As mentioned previously, the patient was invited for this case study in order to understand how balance could improve upon CI stimulation when the vestibular nerve was cut.

### 2.2. Residual vestibular function

Prior to and after the cochlear implantation, several audiovestibular tests were performed. Pre-operative and post-operative tests showed stable results, more specifically, a completely impaired vestibulo-ocular reflex (VOR) during the caloric test (prior to implantation: bilateral bithermal caloric sums = 0°/s) and the sinusoidal harmonic acceleration test (prior to implantation: gain = 0%) (AquaStar and Minitorque, DIFRA, Eupen, Belgium). The caloric test and the sinusoidal harmonic acceleration test were not repeated after cochlear implantation due to the complete areflexia. Similar results of impaired VOR were observed during the video head impulse test (HeadStar, DIFRA, Eupen, Belgium) with pre-operative gains of the right horizontal semicircular canal (hSCC) of 0,42 and a left hSCC gain of 0.53. After the implantation, the right hSCC had a gain of 0.35 and the left SCC had a gain of 0.38. Overt and covert correction saccades were bilaterally present during pre- and post-operative assessment. The results obtained during the three SCC tests were thus in line with the diagnostic criteria for BVP (15).

Saccular function was evaluated by means of air-evoked (insert earphones) cervical vestibular-evoked myogenic potentials (cVEMPs; Neurosoft^®^, NeurAudio^®^, Ivanovo, Russia). The cVEMP was absent on the left but was present and normal on the right before and after the implantation (based on a detection threshold of 115-decibel sound pressure level (dB SPL) before and after implantation, and a corrected amplitude of 0.8 before and 1.3 after the implantation).

The utricular function was assessed by means of vibration-evoked ocular VEMPs (Neurosoft^®^, NeurAudio^®^, Ivanovo, Russia), which were only clinically available in our department after the implantation. The oVEMPs were bilaterally absent during mini shaker stimulation at 121 decibel force level (dB FL) (Mini Shaker type 4810^®^, amplifier model 2718, Brüel & Kjaer^®^, Nærum, Denmark).

As the patient did not report any vertigo attacks since the vestibular neurectomy and as the vestibular assessments (prior to cochlear implantation) showed complete areflexia on the left side, it was assumed that the vestibular neurectomy was complete and that no re-innervation had occurred.

## 3. Diagnostic assessment

### 3.1. Cochlear implant stimulation

Charge-balanced biphasic, cathodic first, pulses were presented through the CI. The stimulation parameters used during the experiment were no different from those used by the patient during daily life. The threshold level and the maximum comfortable level (MCL) were expressed in charge units (Qu), i.e., the charge of one phase which was defined as the product of stimulation current (expressed in current units or cu) and phase duration (expressed in μs). One current unit corresponds with 1 μA. All electrodes had a threshold of at least 1.75 Qu and a maximal MCL of 27.49 Qu. The biphasic pulse width was situated between 17.08 and 25.42 μs (interphase gap = 2.1 μs).

The two stimulation conditions for this study were CI OFF and CI ON. During CI OFF, the participant did not receive electrical stimulation. In the CI ON condition, electrical stimulation was provided by means of the CI, as the environmental sound intensity was 42 dB A, which exceeded the microphone sensitivity of the sound processor (i.e., 30 dB SPL). First, the “CI OFF” condition was performed, followed by the “CI ON” condition.

### 3.2. Posturography

The patient had to stand still on the posturography platform (StabiloPro, DIFRA, Eupen, Belgium) with the feet aligned with shoulder width for 30 s during CI ON and CI OFF in a semi-quiet environment (42 dB A).

The sensors in the platform calculated the following parameters: stability (%), sway area (cm^2^), total path length (cm), and sway velocity (cm/s). These parameters were compared with normative data previously collected in our center (18, 19).

A MATLAB tool (MATLAB and Statistics Toolbox Release 2019a, The MathWorks, Inc., Massachusetts, United States) was used by one of the authors (CDL) for computing the postural sway frequency in the medio-lateral and antero-posterior plane.

### 3.3. Functional Gait Assessment

In addition, the Functional Gait Assessment (FGA) was performed with the CI OFF and ON (in that order) (20). Each FGA task was scored between 0 (“severe impairment”) and 3 (“normal”), and the total score was the sum of all tasks. The minimally detectable change is 6 points in the elderly (60–90 years old) (21).

### 3.4. Statistics and informed consent

Statistical analyses could not be performed because only one patient was evaluated. The study was conducted according to the principles of the Declaration of Helsinki, and written informed consent was obtained from the patient (GZA study number: 181111ACADEM).

## 4. Results

### 4.1. Posturography

In the CI OFF condition, all parameters were outside the normal range. The stability of the patient was 86% (normal range: 88–98%), and the sway velocity was 15 cm/s (normal range: 1–12 cm/s). The sway area (125 cm^2^) was approximately two times the outer bound of the normal range (0–61 cm^2^), and the total path length (447 cm) was outside the normal range (44–360 cm) as well (Table 2).

**Table 2:** Postural stability during cochlear implant activation (“CI ON”) and deactivation (“CI OFF”).

	**CI OFF**	**CI ON**	**MCD (18, 19)**	**Significant improvement based on minimal detectable change?**
Stability (%)	**86**	93	6	Yes
Sway area (cm^2^)	**125**	14	30	Yes
Total path length (cm)	**447**	215	129	Yes
Velocity (cm/s)	**15**	7	4	Yes

Values in bold fell outside the normal range and were thus considered abnormal. MCD, minimal detectable change; CI OFF, cochlear implant (CI) deactivated (no stimulation); CI ON, CI activated.

**Figure 1:**
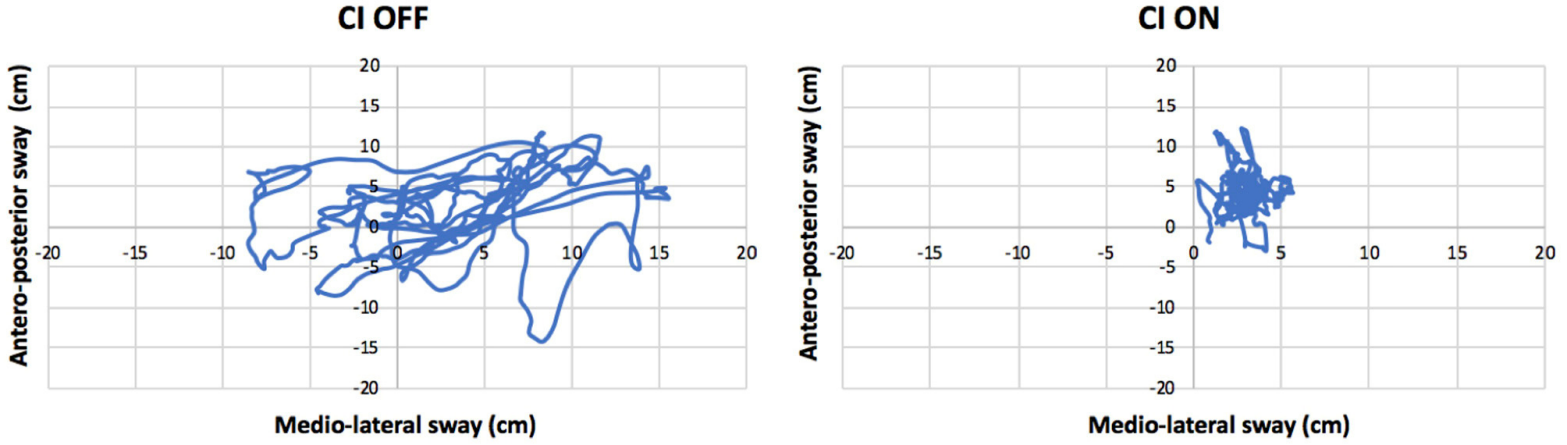
Antero-posterior and medio-lateral swaying (cm) while standing on a stable platform with the eyes closed and cochlear implant (CI) activated (“CI ON”: **right panel**) or deactivated (“CI OFF”: **left panel**).

Turning the CI ON led to an improvement of stability by 7%. The sway area was reduced to 14 cm^2^, and the total path length to 215 cm. The sway velocity was also reduced from 15 cm/s to 7 cm/s. For all these parameters, the minimally detectable change got exceeded (Table 2). The improved stability is illustrated in Figure 1.

**Figure 2:**
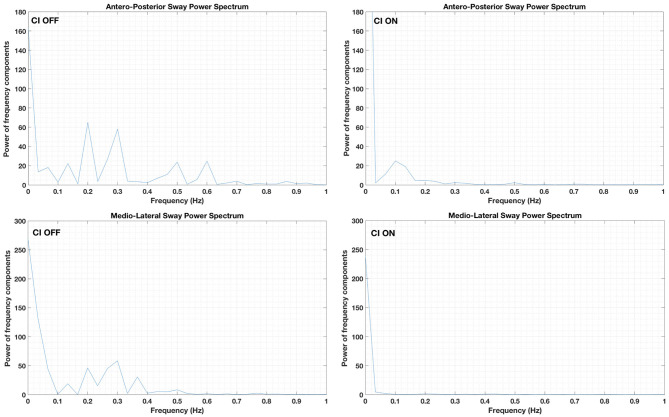
Sway frequency components in the medio-lateral **(lower panels)** and antero-posterior planes **(upper panels)**. Data obtained while the patient stood on a stable platform with eyes closed and the cochlear implant (CI) activated (“CI ON”: **right panels**) or deactivated (“CI OFF”: **left panels**).

The sway frequency changed, depending on the condition (Figure 2). During the CI OFF condition, the power of the fundamental frequency in the medio-lateral sway was 0.29 Hz. Additional frequency components between 0.1 and 0.4 Hz were detected. The power of all these frequency components was reduced when the CI was ON.

In the antero-posterior plane, the fundamental sway frequency was 0.19 Hz followed by an additional frequency component at 0.29 Hz and two smaller frequency components at 0.5 and 0.6 Hz. However, all frequency components were not detected when the CI was ON (Figure 2). A small frequency component at 0.10 Hz was visible when the CI was stimulated; however, the power was limited.

### 4.2. Function Gait Assessment

The total score without CI was 23 out of 30, and the score with CI was 27 out of 30. The difference between both conditions was thus 4 points, which was lower than the minimal detectable change of 6 points.

## 5. Discussion

In this case report, it was described how a patient, who underwent a neurectomy of the left vestibular nerve and BVP reported improved balance whenever she activated her left-sided CI. The results obtained while standing on a stable posturography platform with the eyes closed confirmed that stability (%), sway area (cm^2^), and total path length (cm) clinically significantly improved upon CI activation. As this patient previously underwent a neurectomy of the left vestibular nerve, vestibular co-stimulation as a side effect of the CI stimulation could not be the explanation for the improved balance. In addition, during this experiment, no spontaneous nystagmus was observed (before, during, or after CI stimulation).

A possible explanation for this could be the electrically restored auditory input. The brain requires auditory cues for certain balance-related cognitive tasks such as spatial orientation and spatial learning. Applying this to the CI shows that the CI-mediated auditory reference can contribute to improved vestibular performance. Especially in our patient, who had normal hearing on the right, the combination of unilateral natural hearing with contralateral artificially restored hearing may have contributed to the observed improved balance. Similar results were reported previously by Zhong and Yost (11) and Stevens et al. (12), who investigated the effects of auditory cues on postural balance. Zhong and Yost (11) concluded that auditory cues act similar to an external reference for maintaining balance. For patients who cannot (fully) perceive environmental sounds due to hearing loss, it can be hypothesized that they have more difficulty in maintaining balance, regardless of their peripheral vestibular function. In addition, Stevens et al. (12) reported an inversed relation between the benefit from the auditory signal and the degree of vestibular loss. In other words, the more extensive the vestibular loss, the more benefit from the auditory input. In 2017, Shayman et al. (13) also reported similar results of improved gait when hearing was electrically (CI) or acoustically (hearing aid) restored. The fact that auditory-improved gait and balance are reported in patients with normal hearing or in those with acoustic hearing aids further confirms the hypothesis of auditory cues serving as an external reference for maintaining balance.

A second underlying mechanism that may explain the obtained results is the central inhibition of tinnitus. An accepted theory regarding tinnitus in combination with hearing loss is that it results from maladaptive neuroplasticity in response to the deprived auditory input (16, 17). Auditory deprivation could thus lead to pathological reorganization and altered neural activity. Providing CI stimulation (even at subthreshold level) has been shown to be effective in inhibiting tinnitus (16, 17). A possible explanation for this could be that electrical cochlear stimulation modulates/restores the altered neural activity and reorganization (16, 17). The present patient suffered from chronic tinnitus which could be suppressed by CI stimulation. Deactivating the CI resulted in increased imbalance (as shown above) and the re-occurrence of tinnitus. It may be hypothesized that the presence of tinnitus increases the cognitive load, which is known to be already increased in patients with BVP, leading to reduced performance on static posturography testing. The activation of the CI suppresses tinnitus, possibly facilitating attention and concentration during these tasks.

Nonetheless, no clinically relevant difference was observed regarding the FGA score. A possible explanation for the absence of any clinically significant effects regarding FGA might be the task difficulty, which requires intact fast vestibular reflexes for not losing balance.

A limitation of this study was that during the CI ON condition, only environmental sounds were present (air-conditioning, sound from the computer drivers, …). It would have been interesting to evaluate the effect of different sound conditions (e.g., white noise, babbling, or music). However, the current case report was not written to conclude that using a CI cannot improve balance, but it was written to show that CI stimulation can indeed lead to improved balance but that vestibular co-stimulation was not the reason for it (at least in this case), and this is the reason why the other sound conditions were not included. Moreover, adding sound to the current configuration could have created an external reference for localization in space. It is therefore likely that the balance would have improved as a result of this external reference rather than vestibular co-stimulation.

Next, the possible contribution of a placebo effect should be acknowledged, especially as the patient who entered the experiment was convinced that her balance was better during CI stimulation. This improvement was observed while standing on the posturography platform but not during the FGA. If a placebo effect was present, it should have applied to the FGA as well but no clear difference was observed between CI ON and OFF. Moreover, the FGA performed without CI stimulation was within normal limits. If a placebo effect would have been present, then a worse balance during the FGA (CI OFF) could have been expected due to the fear of falling without the alleged placebo-mediated support of the CI.

An additional possible limitation of this experiment could be the normal contralateral hearing. As this ear was not plugged or masked during the experiment, the measurements during the CI OFF condition may have been influenced by environmental sounds captured by the contralateral ear. However, this set-up allowed simulation of the natural situation during which our subject described impaired balance (i.e., normal hearing on the right and no CI stimulation on the left). As it was the goal to understand how the balance of this specific patient could be improved upon CI activation, it was imperative to recreate an experimental environment that approximated a natural situation according to this subject's standards.

## 6. Conclusion

In this case report, it was described how a patient, who previously underwent a neurectomy of the left vestibular nerve, reported improved balance whenever her CI was in an activated state. This was confirmed by clinically significantly improved sway parameters when the CI was activated. As this patient previously underwent a neurectomy of the left vestibular nerve, vestibular co-stimulation as a side effect of the CI stimulation could not be the explanation. It is more likely that the results can be attributed to the electrically restored auditory input, which serves as an external reference for maintaining balance and spatial orientation. In addition, this patient experienced disturbing tinnitus whenever her CI was deactivated. It is thus plausible that the tinnitus increased her cognitive load, which was already increased because of the BVP, leading to an increased imbalance in the absence of CI stimulation.

## Data availability statement

The raw data supporting the conclusions of this article will be made available by the authors, without undue reservation.

## Ethics statement

The studies involving humans were approved by Commissie Medische Ethiek GZA Ziekenhuizen. The studies were conducted in accordance with the local legislation and institutional requirements. The participants provided their written informed consent to participate in this study. Written informed consent was obtained from the individual(s) for the publication of any potentially identifiable images or data included in this article.

## Author contributions

MS, AZ, LD, and FW contributed to the conception and design of the study. MS, CD, and LD organized the database. MS wrote the first draft of the manuscript. MS, CD, LD, FW, JD, EO, AZ, and CB wrote sections of the manuscript. All authors contributed to the manuscript revision, and read and approved the submitted version.
